# A Statistical Approach Regarding the Diagnosis of Osteoporosis and Osteopenia From DXA: Are We Underdiagnosing Osteoporosis?

**DOI:** 10.1002/jbm4.10444

**Published:** 2021-01-03

**Authors:** Ronnie Sebro, S Sharon Ashok

**Affiliations:** ^1^ Department of Radiology University of Pennsylvania Philadelphia PA USA; ^2^ Department of Orthopaedic Surgery University of Pennsylvania Philadelphia PA USA; ^3^ Department of Genetics University of Pennsylvania Philadelphia PA USA; ^4^ Department of Biostatistics, Epidemiology and Informatics University of Pennsylvania Philadelphia PA USA

**Keywords:** ANALYSIS/QUANTITATION OF BONE, DISEASES AND DISORDERS OF/RELATED TO BONE, DUAL‐ENERGY X‐RAY ABSORPTIOMETRY, OSTEOPOROSIS, STATISTICAL METHODS

## Abstract

Osteoporosis and osteopenia are diagnosed most commonly by evaluating the lowest *T*‐score of BMD measurements, typically taken at three sites: the L1‐L4 lumbar spine, femoral neck, and total hip. This study aimed to evaluate the effect of using all three BMD measurements and multivariate statistical theory to evaluate how the diagnoses of osteoporosis and osteopenia change in simulation studies and in real data. First, it was found that the *T*‐scores from these three BMD measurements rarely give concordant diagnoses using the same World Health Organization (WHO) and International Society for Clinical Densitometry (ISCD) guidelines, so that the diagnosis strongly depends on the BMD sites measured. Next, strong correlations were found between the BMD measurements at different sites within the same person, which resulted in increased congruence/concordance between the diagnoses obtained from the BMD *T*‐scores. Multivariate statistical theory was used to show that the joint distribution of the BMD *T*‐scores at different sites follows a multivariate t distribution and found that the marginal distribution of any BMD *T*‐score follows a univariate t distribution. Confidence ellipsoids were derived that are equivalent to the univariate WHO/ISCD thresholds for osteoporosis (*T*‐score ≤−2.5) and osteopenia (−2.5 < *T*‐score <−1). The study found that more patients are diagnosed with osteoporosis using the multivariate version of the WHO/ISCD guidelines rather than the current WHO/ISCD guidelines in both real data and simulation studies. Diagnoses of osteoporosis using the statistics derived method were also associated with higher FRAX (fracture risk assessment tool) probabilities of major osteoporotic (*p* = 0.001) and hip fractures (*p* = 2.2 × 10^−6^). In conclusion, this study shows that considering all three BMD *T*‐scores is potentially more informative than using the single lowest BMD *T*‐score. © 2020 The Authors. *JBMR Plus* published by Wiley Periodicals LLC. on behalf of American Society for Bone and Mineral Research.

## Introduction

Bone Mineral Density (BMD) is influenced by several factors including genetics, activity, nutrition, and medical comorbidities.^(^ ^1^, ^2^, ^3^ ^)^ BMD naturally decreases after midadulthood and low BMD is associated with increased risk of fractures of the spine, forearm, and hip.^(^ ^1^, ^3^ ^)^ Hip fractures, for example, occur in approximately 400 of 100,000 people in the United States.^(^ ^4^ ^)^ The one‐year mortality following a hip fracture is approximately 21.9% for women and 32.5% for men.^(^ ^4^ ^)^ Therefore, identifying and treating patients with low BMD, who are at risk for fractures, especially hip fractures, is important. Dual‐energy X‐ray absorptiometry (DXA) is the most commonly used screening imaging test for the diagnosis of low BMD.^(^ ^5^, ^6^ ^)^ The BMD is typically measured at three sites: the lumbar spine, specifically, the L1‐L4 vertebral bodies; the femoral neck; and the total hip.^(^ ^6^ ^)^ Osteoporosis in the elderly is diagnosed using the World Health Organization (WHO) and International Society for Clinical Densitometry (ISCD) guidelines when the BMD measured at any site is 2.5 standard deviations (SDs) or more below that of sex‐matched young adults (lowest *T*‐score ≤−2.5); osteopenia in the elderly is diagnosed when the BMD measured at any site is between 1 and 2.5 SDs below that of sex‐matched young adults (−2.5 < lowest *T*‐score <−1).^(^ ^6^, ^7^, ^8^ ^)^ Thus, the WHO/ISCD method uses only one BMD measurement to diagnose osteopenia and osteoporosis.

These three BMD measurements (L1‐L4 BMD, femoral neck BMD, and the total hip BMD) are likely correlated because they are measured within the same individual and they are measuring overall bone homeostasis within that individual. This correlation structure has been largely ignored in clinical practice. For example, a patient with L1‐L4, femoral neck, and total hip BMD *T*‐scores of −1.0, −1.0, and − 1.0 respectively, is normal under the current WHO clinical guidelines, whereas a patient with L1‐L4, femoral neck, and total hip BMD *T*‐scores of −1.1, 1.5, and 1.5 respectively, has osteopenia.^(^ ^7^, ^8^ ^)^ This results in patients with three borderline low‐BMD measurements having a more severe diagnosis than patients with a single marginally low‐BMD measurement.

The current WHO DXA BMD *T*‐score threshold values for the diagnosis of osteoporosis were determined by finding the individual areal BMD *T*‐scores that would give a proportion of individuals osteoporosis that matched the known prevalence of patients with osteoporosis while simultaneously matching the lifetime fracture risk.^(^ ^7^, ^8^, ^9^, ^10^ ^)^ Kanis and colleagues^(7)^ noted that a BMD *T*‐score threshold value of −2.5 identifies approximately 30% of postmenopausal women as having osteoporosis using BMD measurements, and this 30% is approximately equivalent to the lifetime fracture risk (39.7%) at these sites in postmenopausal women (Tables 1 and 2). This is why the threshold *T*‐score of −2.5 was used to define osteoporosis. A prior report noted that this WHO/ISCD definition makes osteoporosis population‐based/statistics‐based rather than BMD‐based.^(^ ^11^ ^)^

**Table 1:** Estimated Lifetime Fracture Risk in White Women (95% CIs) at Age 50 Years^a^

Fracture site	Women
Proximal femur	17.5 (16.8–18.2)
Vertebra	15.6 (14.8–16.3)
Any fragility fracture	39.7 (38.7–40.6)

^a^ Values in the table are derived from Kanis et al^(^ ^7^ ^)^ and Melton 3^rd^ et al.^(^ ^9^ ^)^

**Table 2:** Prevalence of Osteoporosis and Osteopenia in Sweden Using Female‐Derived Thresholds From the Young Population, Aged 20–29 Years^a^

Diagnosis	Age range (years)	Women	
		% of population	No. affected (thousands)
Osteopenia/low bone mass	50–84	49.1	721.3
Osteoporosis	50–84	21.2	310.9
Either osteopenia or osteoporosis	50–84	70.3	1032.2

^a^ Derived from Melton 3^rd^ et al.^(^ ^9^ ^)^

The aim of this study was to use statistical theory to account for the correlations between BMD measurements to create joint thresholds using all three BMD measurements to categorize patients into being osteoporotic or osteopenic. Simulated and real data are used to show the potential change in the number of osteoporosis and osteopenia diagnoses when the multivariate method in which all three BMD measurements are used in the diagnosis of osteopenia and osteoporosis as compared with the current WHO/ISCD method in which only one BMD measurement is used for this diagnosis.

## Patients and Methods

### Statistical theory

First, we show that the joint distribution of the L1‐L4 lumbar spine, femoral neck, and total hip BMD *T*‐scores from DXA studies follow a multivariate T‐distribution.^(^ ^12^, ^13^, ^14^, ^15^, ^16^ ^)^ We calculate 3‐dimensional confidence intervals (3D CIs) from the multivariate T‐distribution to be used for thresholds to diagnose osteopenia and osteoporosis. We show that the marginal distribution of each BMD (L1‐L4 lumbar spine, femoral neck, and total hip) remains a univariate T distribution. We then derive the conditional distribution of any single BMD *T*‐score when the other two BMD *T*‐scores are known (Appendix).

### Simulated data

We simulated random samples of 1000 postmenopausal white women with DXA studies, evaluating the BMD of the L1‐L4 lumbar spine, femoral neck, and total hip (see the Appendix for full details). We assumed that the BMD of the L1‐L4 lumbar spine, the BMD of the femoral neck, and the BMD of the total hip were normally distributed.

We simulated the DXA data of 1000 postmenopausal white women from a trivariate normal distribution with mean, μ_O_,and variance covariance matrix:

$$\Sigma_{O}=\left(\begin{array}{ccc} \sigma_{O.1}^{2} & \rho_{12}\sigma_{O.1}\sigma_{O.2} & \rho_{13}\sigma_{O.1}\sigma_{O.3}\\ \rho_{12}\sigma_{O.1}\sigma_{O.2} & \sigma_{O.2}^{2} & \rho_{23}\sigma_{O.2}\sigma_{O.3}\\ \rho_{13}\sigma_{O.1}\sigma_{O.3} & \rho_{23}\sigma_{O.2}\sigma_{O.3} & \sigma_{O.3}^{2} \end{array}\right),$$

where *ρ*_12_is the correlation between the L1‐L4 BMD and the femoral neck BMD, *ρ*_13_ is the correlation between the L1‐L4 BMD and the total hip BMD, and *ρ*_23_ is the correlation between the femoral neck BMD and the total hip BMD. *σ*_O. 1_ is the SD of the L1‐L4 BMD, *σ*_*O*. 2_ is the SD of the femoral neck BMD, and *σ*_*O*. 3_ is the SD of the total hip BMD. The simulated L1‐L4 BMD, femoral neck BMD, total hip BMD, and the mean and SDs of the young‐adult population L1‐L4 BMD, femoral neck BMD, and total hip BMD (available from the National Health and Nutrition Examination Survey III [NHANES III] cohort for the young‐adult total hip and femoral neck BMD, and from GE Lunar Prodigy DXAs^(10)^ for the young‐adult L1‐L4 BMD) were used to calculate the BMD *T*‐scores in the simulated data.

There were three pairwise correlations between the L1‐L4 BMD, femoral neck BMD, and total hip BMD: (i) the correlation between the L1‐L4 BMD and the femoral neck BMD (*ρ*_12_), (ii) the correlation between the L1‐L4 BMD and the total hip BMD (*ρ*_13_), and (iii) the correlation between the femoral neck BMD and the total hip BMD (*ρ*_23_). We considered five sets of values for these three pairwise correlations, *ρ*_12_, *ρ*_13_, and *ρ*_23_: (0, 0, 0), (0.2, 0.2, 0.2), (0.4, 0.4, 0.4), (0.6, 0.6, 0.6), and (0.8, 0.8, 0.8), respectively, for the simulations.

#### Congruence of simulated BMD measurements

We calculated the proportion of times all three simulated BMD measurements (L1‐L4 BMD, femoral neck BMD, and total hip BMDs) were all congruent and were consistent with a diagnosis of osteoporosis, osteopenia, and normal BMD, respectively, using the WHO guidelines.

#### *Diagnoses of osteoporosis and osteopenia in the simulated data*

From the simulated data, we calculated the proportion of patients that would be diagnosed with osteoporosis, osteopenia, and normal BMD, respectively, using the WHO/ISCD guidelines, for the five different pairwise correlation structures examined.^(^ ^17^ ^)^ We compared these proportions to the proportion of patients that would be diagnosed with osteoporosis, osteopenia, and normal BMD, respectively, using the statistics‐based method.

### Real data

This retrospective study was approved by the local institutional review board (IRB) and the need for signed informed consent from each patient was waived (IRB #832362, date of approval, June 29, 2019). Research carried out with human subjects complied with the World Medical Association Declaration of Helsinki–Ethical Principles for Medical Research Involving Human Subjects. The study was compliant with the Health Insurance Portability and Accountability Act (HIPAA) of 1996. DXA studies were conducted using General Electric (GE) Lunar (Boston, MA, USA; *N* = 674) or Hologic (Marlborough, MA, USA; *N* = 326) DXA machines. DXA studies were performed at a single academic tertiary care institution between January 1, 2019 and January 1, 2020.

The L1‐L4 BMD, femoral neck BMD, and total hip BMD, as well as the L1‐L4 BMD *T*‐scores, femoral neck BMD *T*‐scores, and total hip BMD *T*‐scores were obtained and recorded from a sample of 1000 white women 65 years of age who underwent routine screening DXA studies at a single tertiary‐care academic medical center. Patient age, sex, height, weight, and BMD measurements (BMD and BMD *T*‐scores), history of diabetes mellitus, history of adult fracture, and history of calcium/vitamin D supplementation at the time of the DXA study were recorded. We also recorded the patients who started pharmacologic treatment of osteoporosis.

#### *Congruence of BMD T‐score measurements*

The study sample mean and SDs of the L1‐L4 BMD, femoral neck BMD, and total hip BMD, as well as the mean and SDs of the L1‐L4 BMD *T*‐scores, femoral neck BMD *T*‐scores, and total hip BMD *T*‐scores were calculated. Next, we calculated the proportion of instances where all three BMD *T*‐score measurements were congruent and were consistent with diagnoses of osteoporosis, osteopenia, and normal BMD, respectively, using the WHO/ISCD guidelines.

#### *Diagnoses in the study sample*

The prevalence of osteoporosis, osteopenia, and normal BMD among 65‐year‐old white women in the sample data was estimated using the WHO/ISCD guidelines.^(^ ^17^ ^)^ Then, the prevalence of osteoporosis, osteopenia, and normal BMD among these 65‐year‐old white women in the sample data was estimated using the statistics‐based guidelines.

#### Patients considered for osteoporotic therapy

The fracture risk assessment tool (FRAX) estimates the 10‐year risk of major osteoporosis‐related fractures and hip fractures based on clinical risk factors and BMD measurements obtained at a single site: the femoral neck. Currently, treatment of osteoporosis is considered for patients with osteoporosis based on the WHO guidelines (lowest *T*‐score at any site ≤−2.5) and patients with osteopenia based on the WHO guidelines (lowest *T*‐score at any site between −1.0 and −2.5) with a 10‐year FRAX risk of major osteoporosis‐related fracture of ≥20% or a 10‐year FRAX risk of hip fracture ≥3%. We were asked to evaluate how the number/proportion of patients considered for osteoporotic treatment under the current guidelines would change using our statistics‐based method utilizing all three *T*‐scores. To answer this question, the 10‐year FRAX risk major osteoporosis‐related fracture and 10‐year FRAX risk of hip fracture from the DXA output when performed was collected.

All test statistics were two‐sided. R statistical software was used (R Foundation for Statistical Computing, Vienna, Austria; https://www.r-project.org/).^(^ ^18^ ^)^ The proportions of patients with each diagnosis (normal, osteopenia, and osteoporosis) calculated using the current WHO method and the statistics‐based method were compared using McNemar's test. The type I error rate was set at 0.05, and *p* values <0.05 were considered statistics significant.

## Results

### Simulation study

#### *Congruence of BMD T‐scores*

The mean and SD of each BMD measurement were the same for each site measured, regardless of the correlation structure between BMD measurements at different sites. However, the congruence of the BMD *T*‐score measurements was dependent on the correlation between BMD measurements at different sites. The congruence of the WHO/ISCD diagnoses using BMD *T*‐score measurements increased with higher correlations between the BMD measurements at different sites (Table 3). We found that there was poor congruence between the diagnoses obtained from the BMD measurements at each site using the WHO/ISCD guidelines. Assuming a correlation *r* = 0 between BMD measurements at different sites, 0% (0 of 14) of patients had all three BMD measurements that were congruent for osteoporosis and 1.0% (4of 391) of patients had all three BMD measurements that were congruent for osteopenia. Assuming a correlation *r* = 0.8 between BMD measurements at different sites, 20% (2 of 10) of patients had all three BMD measurements that were congruent for osteoporosis and 21.2% (52 of 245) of patients had all three BMD measurements that were congruent for osteopenia (Table 3). This shows that the congruence of the diagnoses at each site was poor but increases with increasing correlation between the BMD measurements at different sites.

**Table 3:** Simulated Data

Variable	Correlation structure between all three BMD measurements				
	(0, 0, 0)	(0.2, 0.2, 0.2)	(0.4, 0.4, 0.4)	(0.6, 0.6, 0.6)	(0.8, 0.8, 0.8)
Congruence					
No. of patients with all three BMD *T*‐scores congruent for a WHO diagnosis of osteoporosis (%)	0 (0.0%)	0 (0.0%)	0 (0.0%)	0 (0.0%)	2 (0.2%)
No. of patients with all three BMD *T*‐scores congruent for a WHO diagnosis of osteopenia (%)	4 (0.4%)	12 (1.2%)	19 (1.9%)	31 (3.1%)	52 (5.2%)
No. of patients with all three BMD *T*‐scores congruent for a WHO diagnosis of a normal BMD (%)	595 (59.5%)	616 (61.6%)	650 (65.0%)	690 (69.0%)	745 (74.5%)
Diagnoses					
WHO diagnosis of osteoporosis (%)	14 (1.4%)	11 (1.1%)	10 (1.0%)	10 (1.0%)	10 (1.0%)
Statistics‐based diagnosis of osteoporosis (%)	47 (4.7%)	47 (4.7%)	47 (4.7%)	47 (4.7%)	47 (4.7%)
WHO diagnosis of osteopenia (%)	391 (39.1%)	373 (37.3%)	340 (34.0%)	300 (30.0%)	245 (24.5%)
Statistics‐based diagnosis of osteopenia (%)	343 (34.3%)	343 (34.3%)	343 (34.3%)	343 (34.3%)	343 (34.3%)
WHO diagnosis of Normal BMD (%)	595 (59.5%)	616 (61.6%)	650 (65.0%)	690 (69.0%)	745 (74.5%)
Statistics‐based diagnosis of normal BMD (%)	610 (58.1%)	610 (61.0%)	610 (61.0%)	610 (61.0%)	610 (61.0%)

WHO = World Health Organization guidelines used for the diagnosis of osteopenia, osteoporosis, and normal BMD.

(0, 0, 0): correlation between L1‐L4 BMD and femoral neck BMD = 0, correlation between L1‐L4 BMD and total femur BMD = 0, correlation between femoral neck BMD and total femur BMD = 0.

(0.2, 0.2, 0.2): correlation between L1‐L4 BMD and femoral neck BMD = 0.4, correlation between L1‐L4 BMD and total femur BMD = 0.4, correlation between femoral neck BMD and total femur BMD = 0.4.

(0.4, 0.4, 0.4): correlation between L1‐L4 BMD and femoral neck BMD = 0.4, correlation between L1‐L4 BMD and total femur BMD = 0.4, correlation between femoral neck BMD and total femur BMD = 0.4.

(0.6, 0.6, 0.6): correlation between L1‐L4 BMD and femoral neck BMD =0.6, correlation between L1‐L4 BMD and total femur BMD = 0.6, correlation between femoral neck BMD and total femur BMD = 0.6.

(0.8, 0.8, 0.8): correlation between L1‐L4 BMD and femoral neck BMD = 0.8, correlation between L1‐L4 BMD and total femur BMD = 0.8, correlation between femoral neck BMD and total femur BMD = 0.8.

#### *Diagnoses*

**Fig 1:**
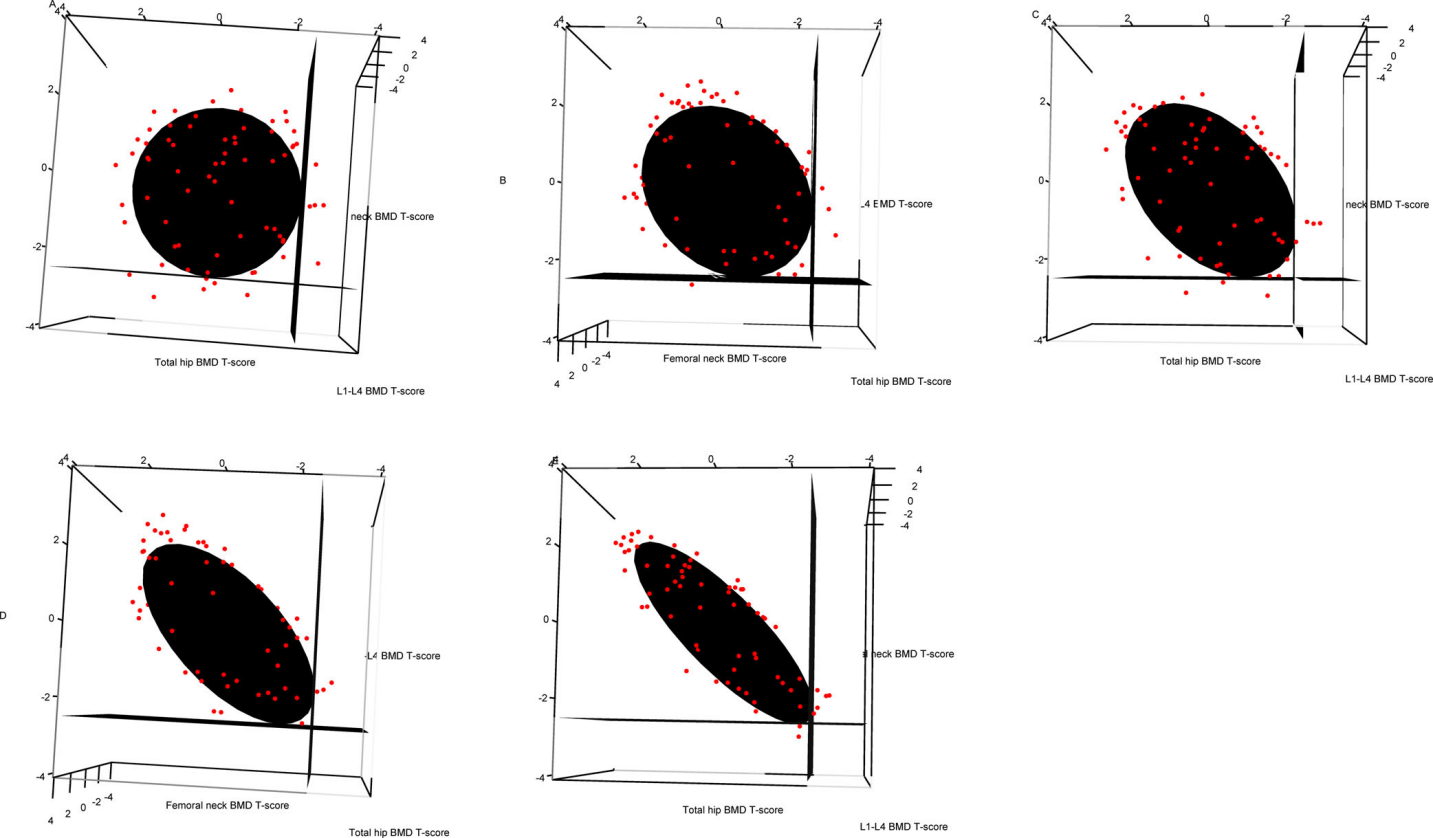
Confidence ellipsoids for the diagnosis of osteoporosis were generated assuming the correlations between BMD measurements (ρ12, ρ13, ρ23) are (0, 0, 0) (Figure 1A), (0.2, 0.2, 0.2) (Figure 1B), (0.4, 0.4, 0.4) (Figure 1C), (0.6, 0.6, 0.6) (Figure 1D), and (0.8, 0.8,0.8) (Figure 1E), respectively.

**Fig 2:**
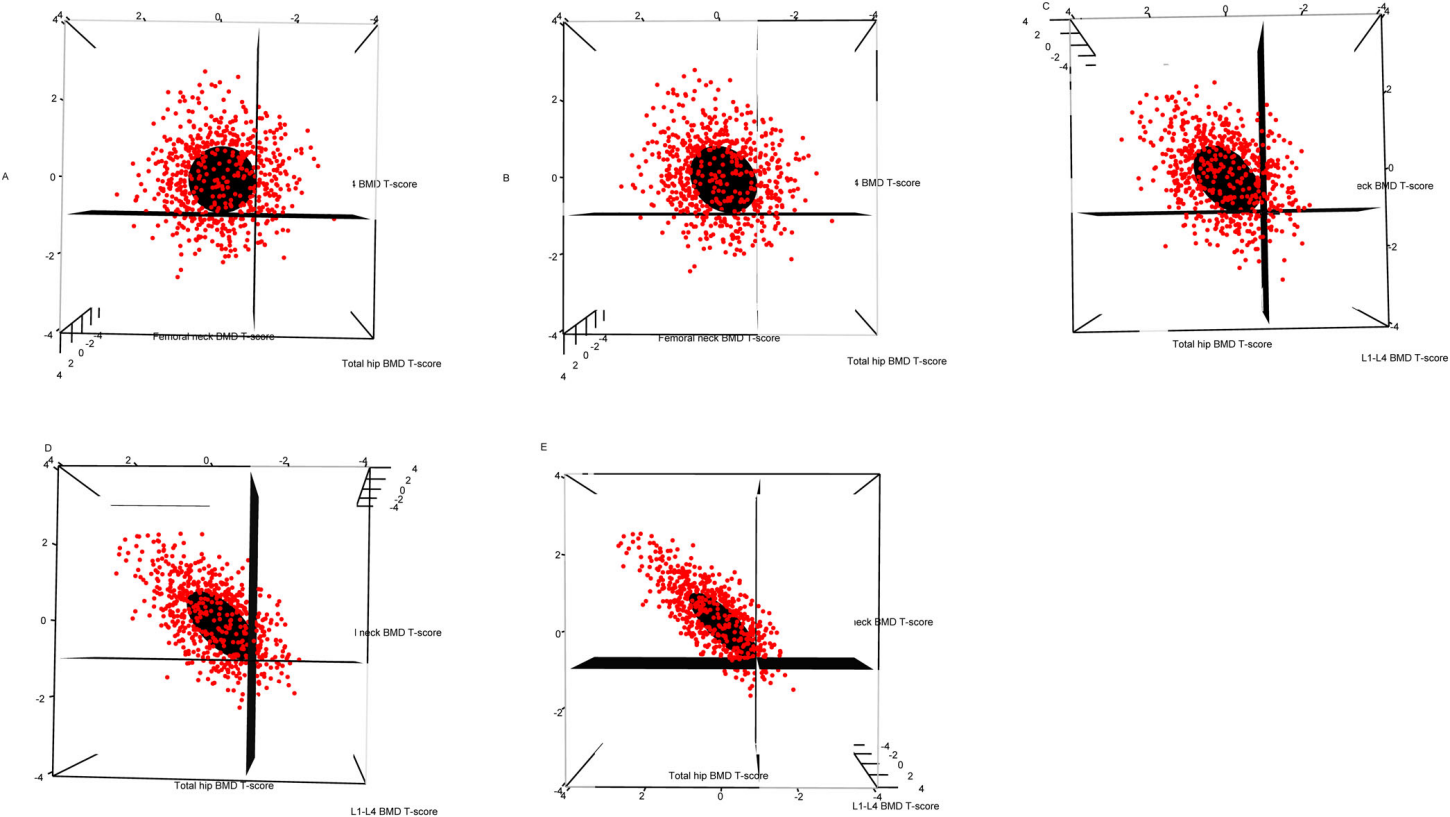
Confidence ellipsoids for the diagnosis of osteopenia in simulated data. Confidence ellipsoids were generated assuming the correlations between BMD measurements (*ρ*_12_, *ρ*_13_, *ρ*_23_) are (*A*) (0, 0, 0), (*B*) (0.2, 0.2, 0.2), (*C*) (0.4, 0.4, 0.4), (*D*) (0.6, 0.6, 0.6), and (*E*) (0.8, 0.8, 0.8), respectively. Simulated data points (red) are superimposed to show the clustering of these data points.

The prevalence of osteoporosis using the WHO/ISCD guidelines decreased with higher correlations between the BMD measurements in the simulated data. We found that there was also an increase in the prevalence of patients with normal BMD using the WHO/ISCD guidelines when there were high correlations between the BMD measurements. However, when using the statistics‐based method, we found that there was no change in the numbers/proportions of patients with osteoporosis and osteopenia regardless of the correlation structure between the BMD measurements (Table 3). Confidence ellipsoids for the diagnosis of osteoporosis were generated assuming the correlations between BMD measurements (ρ12, ρ13, ρ23) are (0, 0, 0) (Figure 1A), (0.2, 0.2, 0.2) (Figure 1B), (0.4, 0.4, 0.4) (Figure 1C), (0.6, 0.6, 0.6) (Figure 1D), and (0.8, 0.8,0.8) (Figure 1E), respectively. Simulated data points are superimposed to show the clustering of these data points. The planes corresponding to *T*‐score thresholds of −2.5 in each of the three axes are included to illustrate the difference between using the WHO/ISCD method and the statistics‐based method. Figure 2 shows the confidence ellipsoids for the diagnosis of osteopenia with superimposed simulated data points assuming the correlations between BMD measurements (ρ12, ρ13, ρ23) are (0, 0, 0) (Figure 2A), (0.2, 0.2, 0.2) (Figure 2B), (0.4, 0.4, 0.4) (Figure 2C), (0.6, 0.6, 0.6) (Figure 2D), and (0.8, 0.8, 0.8) (Figure 2E), respectively. The planes corresponding to *T*‐score thresholds of −1 in each of the three axes are included to illustrate the difference between using the WHO/ISCD guidelines and the statistics‐based method.

### Real data

Most patients (60.3%) were already taking a multivitamin/calcium/vitamin D supplement. There were 24 patients (2.4%) with a prior history of adult fracture before the DXA study and 71 patients (7.1%) with a history of diabetes mellitus. One hundred patients (10.0%) were started on pharmacologic therapy for osteoporosis after the DXA screening test.

#### *Congruence of BMD T‐scores*

The BMD measurements were congruent at all three sites for 41.1% (411 of 1000) of the patients in the study sample. There were 26 patients (2.6%) who had a consistent diagnosis of osteoporosis using the WHO/ISCD guidelines using all three of their BMD measurements (L1‐L4 BMD *T*‐score, femoral neck BMD *T*‐score, and total hip BMD *T*‐score), 18.3% (183 of 1000) of patients had a consistent WHO/ISCD diagnosis of osteopenia using all three of their BMD measurements, whereas 20.2% (202 of 1000) of patients had a consistent diagnosis of normal BMD using all three of their BMD measurements (Table 4). Of the patients with WHO/ISCD osteoporosis, 11.5% (26/226) had all three BMD measurements that were congruent; of the patients with WHO/ISCD osteopenia, 32.0% (183 of 572) had all three BMD measurements that were congruent.

**Table 4:** Real Data From 1000 65‐Year‐Old White Women Undergoing Screening DXA Studies

Variable	Real data
Height in inches (SD)	63.9 (2.6)
Weight in lbs (SD)	156.0 (35.3)
L1‐L4 BMD in g/cm^2^ (SD)	1.03 (0.19)
L1‐L4 BMD *T*‐score (SD)	−0.90 (1.50)
Femoral neck BMD in g/cm^2^ (SD)	0.77 (0.13)
Femoral neck BMD *T*‐score (SD)	−1.54 (0.93)
Total hip BMD in g/cm^2^ (SD)	0.86 (0.13)
Total hip *T*‐score (SD)	−0.97 (1.04)
Diabetes mellitus (*n*, %)	71 (7.1%)
Prior fracture, *n* (%)	24 (2.4%)
Calcium/vitamin D supplementation	603 (60.3%)
Congruence	
No. of patients with all three BMD *T*‐scores congruent for a WHO diagnosis of osteoporosis (%)	26 (2.6%)
No. of patients with all three BMD *T*‐scores congruent for a WHO diagnosis of osteopenia (%)	183 (18.3%)
No. of patients with all three BMD *T*‐scores congruent for a WHO diagnosis of a normal BMD (%)	202 (20.2%)
Total	411 (41.1%)
Diagnoses	
WHO diagnosis of osteoporosis (%)	226 (22.6%)
Statistics‐based diagnosis of osteoporosis (%)	373 (37.3%)
WHO diagnosis of osteopenia (%)	572 (57.2%)
Statistics‐based diagnosis of osteopenia (%)	468 (46.8%)
WHO diagnosis of normal BMD (%)	202 (20.2%)
Statistics‐based diagnosis of normal BMD (%)	159 (15.9%)

#### *Correlations between BMD at different sites*

The estimated correlation between the L1‐L4 BMD and the femoral neck BMD, *r*_12_ was 0.66 (95% CI, 0.62–0.69; *p* < 0.001). The estimated correlation between the L1‐L4 BMD and total hip BMD, *r*_13_ was 0.61 (95% CI, 0.57–0.65; *p* < 0.001). The estimated correlation between femoral neck BMD and total hip BMD, *r*_23_ was 0.75 (95% CI, 0.72–0.78; *p* < .001).

#### *Diagnoses*

**Fig 3:**
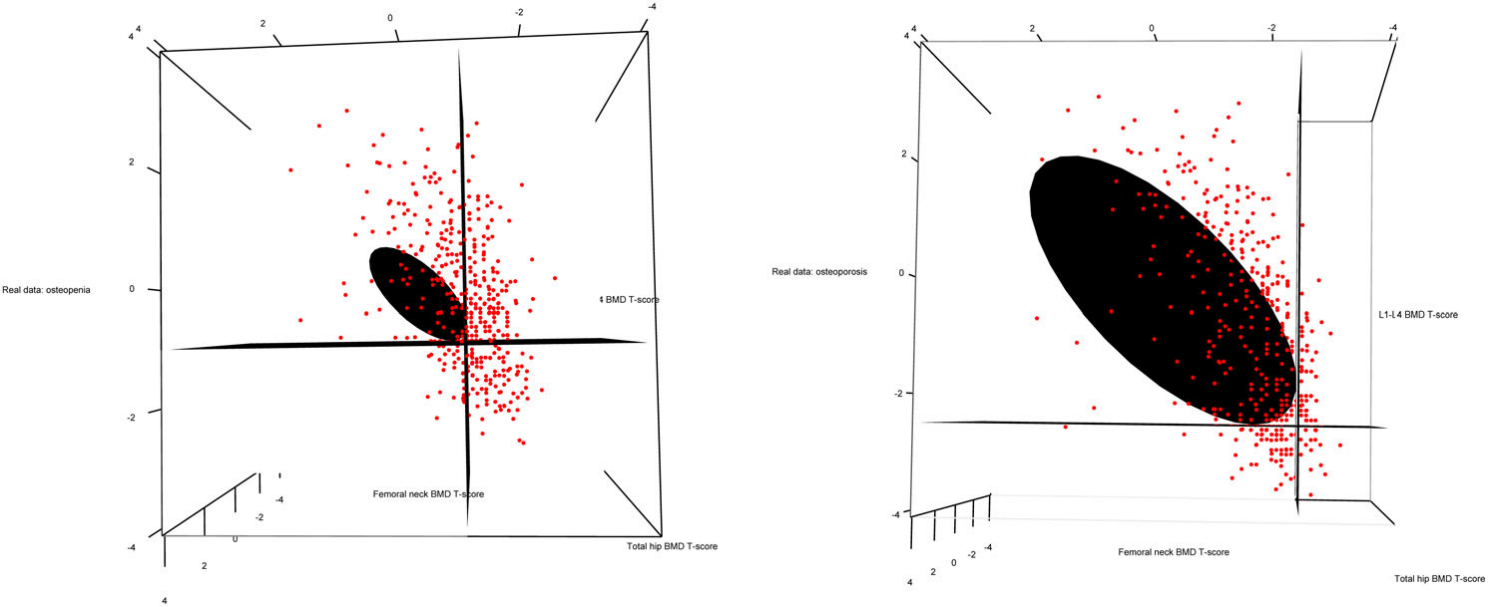
Confidence ellipsoids for the diagnosis of osteoporosis and osteopenia in real data. Confidence ellipsoids were generated assuming the correlations between BMD measurements (*ρ*_12_, *ρ*_13_, *ρ*_23_) were 0.6558, 0.6145, and 0.7508. The first subfigure (*A*) is for the diagnosis of osteopenia. The second subfigure (*B*) is for the diagnosis of osteoperosis. Data points (red) are superimposed to show the clustering of these data points.

Of the study sample, 22.6% (226 of 1000) had osteoporosis and 57.2% (572 of 1000) had osteopenia using the WHO/ISCD guidelines. However, when using the statistics‐based method, we found that approximately 373 patients would be diagnosed with osteoporosis, a 65% increase; and 468 patients would be diagnosed with osteopenia, a 19.2% decrease. Table 4 gives a summary of the clinical and demographic statistics for the 1000 65‐year‐old white women in the study. All patients with WHO/ISCD osteoporosis were diagnosed as having osteoporosis using the statistics‐based method. We found that 79.8% of the sample were osteopenic/osteoporotic using WHO/ISCD guidelines, whereas 84.1% of the sample were osteopenic/osteoporotic using the statistics‐based method. The greatest difference between the statistics‐based method and the WHO/ISCD guidelines was that more patients would be classified as having osteoporosis under the statistics‐based method. McNemar's test showed that the statistics‐based metric identified more patients with osteopenia/osteoporosis than the WHO/ISCD guidelines (χ^2^ = 24.16, *p* < 8.8 × 10^−7^) and the statistics‐based metric identified more patients with osteoporosis than the WHO/ISCD guidelines (χ^2^ = 145.01, *p* < 2.2 × 10^−16^). Confidence ellipsoids for the diagnosis of osteopenia (Figure 3A) and osteoporosis (Figure 3B) with superimposed data points and WHO/ISCD guidelines using the real data. The planes corresponding to *T*‐score thresholds of −2.5 and −1.0 in each of the three axes are included to illustrate the difference between using the WHO/ISCD guidelines and the statistics‐based method on each respective plot.

#### *Patients considered for osteoporotic therapy*

Of the 572 patients with a WHO/ISCD diagnosis of osteopenia, 523 (91.4%) had 10‐year FRAX risk scores. Of these 523 patients with osteopenia and FRAX risk scores, 57 had 10‐year FRAX risk of major osteoporosis‐related fracture of ≥20% or a 10‐year FRAX risk of hip fracture ≥3%; of these 57 patients, 26 (45.6%) would have been classified as osteoporotic using the statistics‐based method. Of the 523 patients with osteopenia, 133 (25.4%) would have been classified as osteoporotic using the statistics‐based method. The patients who would have been classified as osteoporotic using the statistics‐based method had a significantly higher 10‐year FRAX risk of major osteoporosis‐related fracture (13.36%) than those who were not classified as osteoporotic using the statistics‐based method (11.58%, *p* = 0.001). Similarly, the patients who would have been classified as osteoporotic using the statistics‐based method had a significantly higher 10‐year FRAX risk of hip fracture (1.96%) than those who were not classified as osteoporotic using the statistics‐based method (1.42%, *p* = 2.3 × 10^−6^).

## Discussion

DXA studies are routinely used for the measurement of BMD. BMD measurements are typically obtained at three sites: the lumbar spine (L1‐L4 BMD), the femoral neck, and total hip. The current WHO/ISCD guidelines are based on the lowest BMD *T*‐score at any site, and the threshold BMD *T*‐scores for the diagnosis of osteoporosis and osteopenia were determined based on the population prevalence of osteoporosis and osteopenia. Instead of using only the lowest BMD *T*‐score for diagnosis, we considered using all three BMD *T*‐scores in a joint BMD *T*‐score threshold. The *T*‐score thresholds were constrained to −2.5 (for osteoporosis) and −1.0 (for osteopenia) when generating the confidence ellipsoids. Because of this, all patients diagnosed with osteoporosis using WHO/ISCD guidelines would be diagnosed with osteoporosis using the statistics‐based guidelines.

We found that the BMD measurements at different sites were highly correlated. The results of the simulated study suggest that 37 (370%) more patients would be diagnosed with osteoporosis, and 98 (40%) more patients would be diagnosed with osteopenia assuming the correlation between BMD measurements was 0.8, which is closest to what the correlation is likely to be in practice. Our findings suggest that there are individuals with osteoporosis from a statistical viewpoint, who are untreated because they do not satisfy current WHO/ISCD criteria for diagnosis. This potentially has significant clinical implications because the number of individuals eligible for clinical treatment could be much larger than currently thought. Further research is required to determine whether the patients, who would have been diagnosed as osteopenic or osteoporotic using the joint threshold BMD *T*‐score method but normal using the WHO/ISCD guidelines, would have had fewer fragility fractures if they were treated.

The *T*‐scores at all three sites do not always give the same WHO/ISCD diagnosis, and because only the lowest *T*‐score is used, there is information in the other two discarded *T*‐scores that may be clinically relevant. In clinical practice, a patient with L1‐L4, femoral neck, and total hip BMD *T*‐scores of −1.5, 1.5, and 1.5, respectively, on their first study and on a subsequent study has BMD *T*‐scores of −1.5, −1.4, −1.4, has the same diagnosis on both studies despite the changes in the femoral neck and total hip BMD *T*‐scores because only the lowest *T*‐score is considered. Therefore, there is merit in using all three BMD *T*‐scores. The multivariate model can be extended to include the forearm BMD *T*‐scores or any combination of one or more *T*‐scores, including trabecular bone *T*‐scores.

Although the FRAX scores are based on clinical data and only a single BMD measurement, we showed that there was a strong association between FRAX scores and the statistics‐based method for the diagnosis of osteoporosis. This shows that all BMD measurements are more predictive of fracture risk rather than just the BMD at a single site: the femoral neck for patients with osteopenia using the WHO/ISCD guidelines. Further research is required to evaluate how the FRAX scores could be improved using all three BMD measurements.

There are a few limitations regarding this analysis. The true correlation structure between DXA measurements in young adults is unknown and was simulated in our simulated data. The correlation structure between BMD measurements in the real patient data may not generalize to all ages and sexes. Our method captures information that is likely clinically relevant but ignored in typical interpretation of DXA studies. In particular, the correlation structure between DXA measurements of BMD at different sites (L1‐L4, femoral neck, and total hip) are likely clinically relevant. For example, it is known that corticosteroid use preferentially affects and decreases lumbar spine BMD relative to the femoral neck and total hip BMD.^(^ ^19^, ^20^ ^)^ Therefore, the correlation structure between BMD measurements at different sites for patients treated with corticosteroids is likely different from that in the young, untreated adult population. Other studies have shown that the DXA studies provide valuable data that can be used to identify osteoblastic metastases and spinal fractures through changes in this correlation structure along with other DXA measurements.^(^ ^20^, ^21^, ^22^ ^)^ The mean and SD BMDs for the femoral neck and total hip were obtained from the NHANES III cohort, but each DXA manufacturer uses slightly different mean and SD L1‐L4 BMDs because this measurement was not obtained in the NHANES III cohort. This may influence the correlation structure noted between the L1‐L4 BMD and the femoral neck BMD, and total hip BMD measurements. A limitation of the real data analysis was that patients were evaluated using different DXA machines. Although the DXA machines were calibrated according to the International Society for Clinical Densitometry (ISCD) guidelines, there is the possibility that variability between machines may have affected the results. Finally, we acknowledge that the WHO/ISCD guidelines use the lowest BMD *T*‐score, which has been validated in clinical practice; however, it remains unclear whether the site with the lowest BMD *T*‐score is the site at highest risk for a fragility fracture and whether the lowest BMD measurement is more globally informative than all three BMD measurements.

Although we use the term “statistics‐based model,” it is important to note that the current WHO/ISCD guidelines are in fact statistics‐based but univariate because they use only one BMD *T*‐score, as noted by Elandt‐Johnson and colleagues.^(^ ^11^ ^)^ Our model is a more sophisticated multivariate statistical method used to diagnose osteoporosis and osteopenia.

In summary, the proportion of patients diagnosed with osteoporosis from DXA studies using one BMD *T*‐score under current WHO/ISCD guidelines is less than the proportion of patients diagnosed with osteoporosis using a statistics‐based multivariate method in which all three BMD *T*‐scores are used. Further research needs to be performed to assess whether the multivariate method predicts fracture risk better than the univariate method.

## Disclosures

The authors declare no conflicts of interest.

### Peer Review

The peer review history for this article is available at https://publons.com/publon/10.1002/jbm4.10444.

## Data Availability

Data are freely available upon request from the corresponding author.

## Appendix

Statistical derivation showing the joint distribution of BMD *T*‐scores is a multivariate T distribution

Assume ***Y***_***O***_ ***=*** (Y_*O*1_, Y_*O*2_, Y_*O*3_), ***μ***_***O***_ ***=*** (***μ***_***O*1**_, ***μ***_***O*2**_, ***μ***_***O*3**_), ***Y***_***Y***_ ***=*** (Y_*Y*1_, Y_*Y*2_, Y_*Y*3_), ***μ***_***Y***_ ***=*** (***μ***_***Y*1**_, ***μ***_***Y*2**_, ***μ***_***Y*3**_), and **0* =*** **(0,0,0)** are 3 × 1 vectors, and that Σ_*O*_ and Σ_*Y*_ are 3 × 3 positive definite symmetric matrices.

Let *Y*_*O*1_ be the L1‐L4 BMD, *Y*_*O*2_ be the femoral neck BMD, and *Y*_*O*3_ be the total hip BMD of a 65‐year‐old white woman. Assume that *Y*_*O*1_, *Y*_*O*2_, and *Y*_*O*3_ are each normally distributed. Then, *Y*_*O*1_, *Y*_*O*2_, and *Y*_*O*3_ are jointly distributed as a multivariate normal distribution with mean *μ*_*O*_ = (*μ*_*O*1_, *μ*_*O*2_, *μ*_*O*3_) and variance–covariance matrix.

$$\Sigma_{O}=\left(\begin{array}{ccc} \sigma_{O1}^{2} & \rho_{12}\sigma_{O1}\sigma_{O2} & \rho_{13}\sigma_{O1}\sigma_{O3}\\ \rho_{12}\sigma_{O1}\sigma_{O2} & \sigma_{O2}^{2} & \rho_{23}\sigma_{O2}\sigma_{O3}\\ \rho_{13}\sigma_{O1}\sigma_{O3} & \rho_{23}\sigma_{O2}\sigma_{O3} & \sigma_{O3}^{2} \end{array}\right),$$

where *Y*_*O*1_ ∼N(*μ*_*O*1_, σ_*O*1_), *Y*_*O*2_ ∼N(*μ*_*O*2_, σ_*O*2_), *Y*_*O*3_ ∼ N(*μ*_*O*3_, σ_*O*3_), and *ρ*_12_ is the correlation between the L1‐L4 BMD and femoral neck BMD (*Y*_*O*1_ and *Y*_*O*2_), *ρ*_13_ is the correlation between the L1‐L4 BMD and total hip BMD (*Y*_*O*1_ and *Y*_*O*3_), and *ρ*_23_ is the correlation between the femoral neck BMD and total hip BMD (*Y*_*O*2_ and *Y*_*O*3_). Let ***X***_***O***_ ***=*** ( ***Y***_***O***_ ***− μ***_***O***_), then, ***X***_***O***_***∼MVN***(**0**, **Σ**_***O***_).

Let *Y*_*Y*1*i*_ be the L1‐L4 BMD, *Y*_*Y*2*i*_ be the femoral neck BMD, and *Y*_*Y*3*i*_ be the total hip BMD of the *i*th normal 20‐ to 29‐year‐old white woman in the NHANES III data. Estimates of the mean and SDs of the National Health and Nutrition Examination Survey (NHANES III) young non‐Hispanic white women aged 20 to 29 years, femoral neck BMD, and total hip BMD were calculated from the *T*‐scores.^(^ ^10^ ^)^ NHANES III participants were excluded from the DXA studies if they reported radiographic contrast material (barium) use within the past 7 days or nuclear medicine studies within the past 3 days, or if they weighed over 300 pounds or were taller than 6 feet 5 inches. The L1‐L4 BMD was not obtained as part of NHANES III.^(^ ^10^ ^)^ Instead, each DXA scanner manufacturer provided its own estimates of the L1‐L4 BMD in the young adult population. The mean and SDs of the L1‐L4 BMD in the young adult population were also derived from the L1‐L4 BMD *T*‐scores similarly.

Assume that *Y*_*Y*1*i*_, *Y*_*Y*2*i*_, and *Y*_*Y*3*i*_ are normally distributed. Then, *Y*_*Y*1*i*_, *Y*_*Y*2*i*_, and *Y*_*Y*3*i*_ are jointly distributed as a multivariate normal distribution with mean *μ*_*Yi*_ = (*μ*_*Y*1*i*_, *μ*_*Y*2*i*_, *μ*_*Y*3*i*_) and variance–covariance matrix

$$\Sigma_{Y}=\left(\begin{array}{ccc} \sigma_{Y1}^{2} & \rho_{12}^{\prime }\sigma_{Y1}\sigma_{Y2} & \rho_{13}^{\prime }\sigma_{Y1}\sigma_{Y3}\\ \rho_{12}^{\prime }\sigma_{Y1}\sigma_{Y2} & \sigma_{Y2}^{2} & \rho_{23}^{\prime }\sigma_{Y2}\sigma_{Y3}\\ \rho_{13}^{\prime }\sigma_{Y1}\sigma_{Y3} & \rho_{23}^{\prime }\sigma_{Y2}\sigma_{Y3} & \sigma_{Y3}^{2} \end{array}\right),$$

where *Y*_*Y*1*i*_∼N(*μ*_*Y*1_, σ_*Y*1_), *Y*_*Y*2*i*_∼*N*(*μ*_*Y*2_, σ_*Y*2_), and *Y*_*Y*3*i*_∼*N*(*μ*_*Y*3_, σ_*Y*3_), and where $\rho_{12}^{\prime }$ is the correlation between the L1‐L4 BMD and femoral neck BMD (*Y*_*Y*1*i*_ and *Y*_*Y*2*i*_), $\rho_{13}^{\prime }\;$ is the correlation between the L1‐L4 BMD and total hip BMD (*Y*_*Y*1*i*_ and *Y*_*Y*3*i*_) and $\rho_{23}^{\prime }\;$ is the correlation between the femoral neck BMD and the total hip BMD (*Y*_*Y*2*i*_ and *Y*_*Y*3*i*_). Assume that the correlation structure between the BMD measurements in the 20‐ to 29‐year‐olds is unchanged in the older populations, so that $\left(\rho_{12}^{\prime },{\;\rho }_{13}^{\prime },{\;\rho }_{23}^{\prime }\right)$ = (*ρ*_12_, *ρ*_13_, *ρ*_23_). The values of ***μ***_***Y***_ and Σ_*Y*_ are unknown for young white women aged 20‐ to 29‐years‐old, and were estimated from the NHANES III sample of young white women 20‐ to 29‐years‐old (409 subjects) for the femoral neck and the total hip BMD. The NHANES III sample mean femoral neck BMD, ${\bar{Y}}_{Y2}=\frac{1}{N}\sum_{i=1}^{N}Y_{Y2i}\;$ was 1.052 g/cm^2^, and the sample mean total hip BMD is ${\bar{Y}}_{Y3}=\frac{1}{N}\sum_{i=1}^{N}Y_{Y3i}$, (1.016 g/cm^2^). The estimated mean L1‐L4 BMD, ${\bar{Y}}_{Y1}$ from GE Lunar is 1.176 g/cm^2^.

Let ${\bar{Y}}_{Y}=\left({\bar{Y}}_{Y1},{\bar{Y}}_{Y2},{\bar{Y}}_{Y3}\right)$, *Z*_*Y*1*i*_ = ($Y_{Y1i}-{\bar{Y}}_{Y1}$), *Z*_*Y*2*i*_ = ($Y_{Y2i}-{\bar{Y}}_{Y2}$), *Z*_*Y*3*i*_ = ($Y_{Y1i}-{\bar{Y}}_{Y3}$), *Z*_*Yi*_ = (*Z*_*Y*1*i*_, *Z*_*Y*2*i*_, *Z*_*Y*3*i*_), ${\bar{Z}}_{Y}=\left({\bar{Z}}_{Y1},{\bar{Z}}_{Y2},{\bar{Z}}_{Y3}\right)$, and let $S=\frac{1}{N-1}\left(Z_{\mathrm{Yi}}-{\bar{Z}}_{Y}\right){\left(Z_{\mathrm{Yi}}-{\bar{Z}}_{Y}\right)}^{T}$. Then (*N* − 1)*S*∼*W*_3_( *N* − 1, Σ_*Y*_), where W is a Wishart distribution, and *S* is independent of *X*_*O*_.

$$f\left(S;N-1,\Sigma_{Y}\right)=\frac{{\left|\left(N-1\right)S\right|}^{\frac{\left(N-1\right)-3-1}{2}}}{2^{\frac{\left(N-1\right)3}{2}}{\left|\Sigma_{Y}\right|}^{\frac{\left(N-1\right)}{2}}\Gamma \left(\frac{N-1}{2}\right)}e^{\left[-\frac{1}{2}\mathrm{tr}\left(\Sigma_{Y}^{-1}S\left(N-1\right)\right)\right]}$$

where $\mathrm{tr}\left(\Sigma_{Y}^{-1}S\left(N-1\right)\right)$ is the sum of the diagonal elements of the matrix; ∣(*N* − 1) S∣ is the determinant of the square matrix, (*N* − 1) *S*.

$${\left(N-1\right)}^{\frac{-1}{2}}\;\left[\left(N-1\right)S\right]{\left(N-1\right)}^{\frac{-1}{2}}∼\chi_{N-1}^{2}.$$

Consider a random variable X that has a gamma distribution, Gam (α, β), with shape parameter α, and scale parameter β, so that α > 0 and β > 0. A chi‐squared distribution with ν degrees of freedom (ν > 0) can be written as $\chi_{\upsilon }^{2}=\mathrm{Gam}\left(\frac{\upsilon }{2},\frac{1}{2}\right).$

$f\left(x;\alpha ,\beta \right)=\left\{\begin{array}{c} \frac{\beta^{\alpha }}{\Gamma \left(\alpha \right)}\\ 0 \end{array}\right.x^{\alpha }e^{-\mathrm{\beta x}}$, x > 0

where $\Gamma \left(\alpha \right)=\int_{0}^{\infty }e^{-x}x^{\alpha -1}\mathrm{dx}$

$\frac{S}{N-1}∼\frac{\chi_{N-1}^{2}}{N-1}$. Therefore, $\frac{S}{N-1}∼\text{Gamma}\left(\frac{N-1}{2},\frac{N-1}{2}\right)$.

Under the null hypothesis, $\left(\begin{array}{c} \mu_{Y1}\\ \mu_{Y2}\\ \mu_{Y3} \end{array}\right)=\left(\begin{array}{c} \mu_{O1}\\ \mu_{O2}\\ \mu_{O3} \end{array}\right)$, and $\left(\rho_{12}^{\prime },\;\rho_{13}^{\prime },{\;\rho }_{23}^{\prime }\right)$ = (*ρ*_12_, *ρ*_13_, *ρ*_23_), so that (*Y*_*O*_ − *μ*_*Y*_)∼*MVN*(**0**, Σ_*Y*_)

(1) $$T=\sqrt{N-1}{\left(Y_{0}-\mu_{Y}\right)}^{T}S^{-1}∼\mathrm{MVT}\left(0,\Sigma_{Y},N-1\right)$$

### A.1 Confidence ellipsoids

$$\left(Y_{O}-\mu_{Y}\right)∼\mathrm{MVN}\left(0,\Sigma_{Y}\right)$$

$${\left(Y_{O}-\mu_{Y}\right)}^{T}\Sigma_{Y}^{-1}\left(Y_{O}-\mu_{Y}\right)∼\chi_{3}^{2}$$

$$d\left[{\left(Y_{O}-\mu_{Y}\right)}^{T}\Sigma_{Y}^{-1}\left(Y_{O}-\mu_{Y}\right)/d\right]∼\chi_{3}^{2}$$

Consider,

$${\left(\frac{\left(Y_{O}-\mu_{Y}\right)}{\sqrt{\frac{S}{N-1}}}\right)}^{T}\Sigma_{Y}^{-1}\left(\frac{\left(Y_{O}-\mu_{Y}\right)}{\sqrt{\frac{S}{N-1}}}\right)$$

Then,

$$\frac{d\left[{\left(Y_{O}-\mu_{Y}\right)}^{T}\Sigma_{Y}^{-1}\left(Y_{O}-\mu_{Y}\right)/d\right]}{S/\left(N-1\right)}∼\frac{d\chi_{d}^{2}/d}{\chi_{N-1}^{2}/\left(N-1\right)}$$

$$\frac{\chi_{d}^{2}/d}{\chi_{N-1}^{2}/\left(N-1\right)}∼\mathrm{dF}\left(d,N-1\right)$$

Therefore,

$T\Sigma_{Y}^{-1}T\leq d*{\text{threshold}}_{p}$, where, $\int_{0}^{{\text{threshold}}_{p}}F\;\left(d,N-1\right)=P/100$ and d = 3.

The threshold statistic (*d* * *threshold*_*p*_) that corresponds to a *T*‐score of −2.5 is 6.25. The threshold statistic that corresponds to a *T*‐score of −1 is 1. The axes of the confidence thresholds are the eigenvectors of the variance–covariance matrix. An online tool to evaluate the statistics‐based diagnosis based on user‐provided BMD *T*‐scores is available at http://www.ronniesebro.com/dxa/.

### A.2 Marginal distribution of the multivariate T distribution

Let *T* = (*T*_1_, *T*_2_, *T*_3_), where T has a multivariate distribution with υ degrees of freedom.

$$p\left(T\right)=p\left(\begin{array}{c} T_{1}\\ T_{2}\\ T_{3} \end{array}\right)=\mathrm{MVT}\left(\left(\begin{array}{c} T_{1}\\ T_{2}\\ T_{3} \end{array}\right);\left(\begin{array}{c} 0\\ 0\\ 0 \end{array}\right);\left(\begin{array}{ccc} 1 & \rho_{12}^{\prime } & \rho_{13}^{\prime }\\ \rho_{12}^{\prime } & 1 & \rho_{23}^{\prime }\\ \rho_{13}^{\prime } & \rho_{23}^{\prime } & 1 \end{array}\right);\upsilon \right)$$

The marginal probability density function (pdf) of T_1_, T_2_, or T_3_ can be obtained by applying a linear transformation. For example, consider a column vector *A* = (0, 0, 1). Let *Z* = *AT*.

Then Z has a MVT distribution,

$$p\left(Z\right)=p\left(\begin{array}{c} Z_{1}\\ Z_{2}\\ Z_{3} \end{array}\right)=\mathrm{MVT}\left(\left(\begin{array}{c} Z_{1}\\ Z_{2}\\ Z_{3} \end{array}\right);\left(\begin{array}{c} 0\\ 0\\ 0 \end{array}\right);A\left(\begin{array}{ccc} 1 & \rho_{12}^{\prime } & \rho_{13}^{\prime }\\ \rho_{12}^{\prime } & 1 & \rho_{23}^{\prime }\\ \rho_{13}^{\prime } & \rho_{23}^{\prime } & 1 \end{array}\right)A^{T};\upsilon \right)$$

and $p\left(Z\right)=p\left(\begin{array}{c} 0\\ 0\\ T_{3} \end{array}\right)=p\left(T_{3}\right)=T\left(T_{3};\left(\begin{array}{c} 0\\ 0\\ 0 \end{array}\right);1;\upsilon \right)$

Therefore, the marginal distribution of a multivariate T distribution is a univariate T‐distribution.

### A.3 Conditional distribution of the multivariate T distribution

The variance–covariance matrix of the femoral neck BMD and total hip BMD in the NHANES III young adult female cohort and the L1‐L4 BMD, Σ_*Y*_ can be partitioned into a 2 × 2 matrix:

$$\Sigma_{Y}=\left(\begin{array}{ccc} \sigma_{Y1}^{2} & \rho_{12}^{\prime }\sigma_{Y1}\sigma_{Y2} & \rho_{13}^{\prime }\sigma_{Y1}\sigma_{Y3}\\ \rho_{12}^{\prime }\sigma_{Y1}\sigma_{Y2} & \sigma_{Y2}^{2} & \rho_{23}^{\prime }\sigma_{Y2}\sigma_{Y3}\\ \rho_{13}^{\prime }\sigma_{Y1}\sigma_{Y3} & \rho_{23}^{\prime }\sigma_{Y2}\sigma_{Y3} & \sigma_{Y3}^{2} \end{array}\right)=\left(\begin{array}{cc} \Sigma_{11} & \Sigma_{12}\\ \Sigma_{21} & \Sigma_{22} \end{array}\right),$$

where,

$$\Sigma_{11}=\sigma_{Y1}^{2}$$

$$\Sigma_{22}=\left(\begin{array}{cc} \sigma_{Y2}^{2} & \rho_{23}^{\prime }\sigma_{Y2}\sigma_{Y3}\\ \rho_{23}^{\prime }\sigma_{Y2}\sigma_{Y3} & \sigma_{Y3}^{2} \end{array}\right)$$

$$\Sigma_{21}=\Sigma_{12}^{T}=\left(\begin{array}{c} \rho_{12}^{\prime }\sigma_{Y1}\sigma_{Y2}\\ \rho_{13}^{\prime }\sigma_{Y1}\sigma_{Y3} \end{array}\right)$$

The conditional probability density function is given by,

$$p\left(T_{1}|T_{2}=t_{2},T_{3}=t_{3}\right)=\frac{p\left(T\right)}{p\left(T_{2},T_{3}\right)}=\mathrm{MVT}\left(T_{1};\mu_{1|2,3};\Sigma_{1|2,3};\nu_{1|2,3}\right)$$

where,

$$\mu_{1|2,3}=0+\Sigma_{12}\Sigma_{22}^{-1}\left(\left(\begin{array}{c} t_{2}\\ t_{3} \end{array}\right)-\left(\begin{array}{c} 0\\ 0 \end{array}\right)\right)$$

$$\Sigma_{1|2,3}=\frac{\nu +{\left(\left(\begin{array}{c} t_{2}\\ t_{3} \end{array}\right)-\left(\begin{array}{c} 0\\ 0 \end{array}\right)\right)}^{T}\Sigma_{22}^{-1}\left(\left(\begin{array}{c} t_{2}\\ t_{3} \end{array}\right)-\left(\begin{array}{c} 0\\ 0 \end{array}\right)\right)}{\nu +2}\left(\Sigma_{11}-\Sigma_{12}\Sigma_{22}^{-1}\Sigma_{12}^{T}\right)$$

$$\nu_{1|2,3}=\nu +2$$
